# Steady improvement of infection control services in six community hospitals in Makkah following annual audits during Hajj for four consecutive years

**DOI:** 10.1186/1471-2334-6-135

**Published:** 2006-08-25

**Authors:** Tariq A Madani, Ali M Albarrak, Mohammad A Alhazmi, Tarik A Alazraqi, Abdulahakeem O Althaqafi, Abdulrahman H Ishaq

**Affiliations:** 1 Department of Medicine, Faculty of Medicine, King Abdulaziz University, Jeddah, Kingdom of Saudi Arabia; 2 Ministry of Health, Riyadh, Kingdom of Saudi Arabia; 3 Department of Medicine, Armed Forces Hospital, Riyadh, Kingdom of Saudi Arabia; 4 Department of Medicine, Faculty of Medicine, King Khalid University, Abha, Kingdom of Saudi Arabia; 5 Department of Medicine, King Abdulaziz Medical City, Jeddah, Kingdom of Saudi Arabia

## Abstract

**Background:**

the objective of this study was to evaluate the impact of annual review of the infection control practice in all Ministry of Health hospitals in the holy city of Makkah, Saudi Arabia, during the Hajj period of four lunar Islamic years, 1423 to 1426 corresponding to 2003 to 2006.

**Methods:**

audit of infection control service was conducted annually over a 10-day period in six community hospitals with bed capacities ranging from 140 to 557 beds. Data were collected on standardized checklists on various infection control service items during surprise visits to the medical, pediatric, surgical, and critical care units, and the kitchens. Percentage scores were calculated for audited items. The results of the audit for hospitals were confidentially sent to them within four weeks after the end of Hajj.

**Results:**

deficiencies observed in the first audit included lack of infection control committees, infection control units, infection control educational activities, and surveillance system and shortage of staff. These deficiencies were resolved in the subsequent audits. The average (range) scores of hospitals in 11 infection control items increased from 43% (20–67%) in the first audit to 78% (61–93%) in the fourth audit.

**Conclusion:**

regular hospital infection control audits lead to significant improvement of infection control practice. There is a need to build a rigorous infection control audit into hospitals' ongoing monitoring and reporting to the Ministry of Health and to provide these hospitals with feed back on such audits to continuously strengthen the safety standards for patients, visitors, and employees.

## Background

Two to three million pilgrims gather in Makkah annually in the twelfth month of the lunar Islamic year to perform Hajj, the fifth pillar of Islam. During the Rift Valley fever epidemic that occurred in southwestern Saudi Arabia in 2000–2001, a total of 886 cases were reported with 13.9% mortality rate [1]. The infection was mainly transmitted by mosquito bites and/or direct contact with infected sheep [1]. Even though no cases were reported from the holy city of Makkah, there was a potential for its transmission in this city because hundreds of thousands of sheep are sacrificed by pilgrims as part of the Islamic rituals of Hajj. On the other hand, 37 cases of a novel viral hemorrhagic fever virus, referred to as Alkhumra virus, were reported solely from Makkah in 2001–2002, and the virus was also believed to be transmitted by mosquito bites and/or direct contact with infected sheep [2]. After the emergence of these two diseases, the infection control practice in Makkah hospitals during Hajj was scrupulously reviewed by the Saudi Ministry of Health (MOH) to ensure the highest infection control standards for pilgrims. A committee was formed for that purpose comprising consultants in infectious diseases and infection control from the Ministry of Health, the medical schools in King Abdulaziz, King Saud, and King Khaled universities, the Armed Forces Hospital, and the National Guard Hospital. An audit tool was developed by the committee and used to review the infection control practice in all MOH hospitals in Makkah during the Hajj period for four consecutive years. This study describes the results and the impact of this audit on infection control practice in the audited hospitals.

## Methods

### The Hajj

Hajj is the fifth of the five pillars of Islam. Any healthy Muslim adult is obliged to perform Hajj once in his/her life if he/she is financially and physically capable. The Hajj begins on the 8^th ^day of Dhul-Hijjah, the 12^th ^month of the lunar Islamic year, and ends on the 13^th ^day of the same month. Hajj has to be performed in three main locations in Makkah, namely, the sacred Kaaba (in the holy city of Makkah), and Mena and Arafat, which are approximately 5 and 18 Kilometers far from Makkah, respectively. Approximately, 2–3 million pilgrims perform Hajj every year; one third of them come from within Saudi Arabia and two thirds come from other countries. Most pilgrims stay in fire-resistant air-conditioned camping tents in Mena during the entire Hajj period. Financially deprived pilgrims who can not afford to pay for the cost of staying in camps usually stay outdoor. Free medical care services are provided to pilgrims by the Saudi Ministry of Health.

### Study period

The study was conducted over a 10-day period annually for four consecutive years during the Hajj period of the lunar Islamic year 1423 to 1426, corresponding to 2003 to 2006.

### Audited Makkah hospitals

All MOH hospitals in Makkah were included in the audit, namely, Ajiad General Hospital (AGH), Alnoor Specialist Hospital (NSH), King Abdulaziz Hospital (KAH), King Faisal Hospital (KFH), the Maternity and Children Hospital (MCH), and Heraa General Hospital (HGH). Clinical services in AGH, HGH, KAH, KFH, and NSH included internal medicine, general surgery, orthopedic surgery, obstetrics and gynecology, pediatrics, critical care, ophthalmology, and ear, nose, and throat. In addition, the HGH had a neurosurgical service, and the NSH had both neurosurgical and vascular surgery services. The clinical services in the MCH included general pediatrics, neonatal and pediatric critical care, and obstetrics and gynecology. In addition to serving the population of Makkah, these six tertiary care hospitals provide medical care to pilgrims who come to Makkah to perform Hajj during the Hajj period and those who come to Makkah year-round to perform Omra which is similar to Hajj except for the fact that the pilgrims are not required to stay in Mena and Arafat and that there is no specified period of time to perform it. The Hajj period is considered to be a peak-period where additional health care workers are temporarily recruited mainly from other regions in Saudi Arabia and a few from outside the country to cover the extensive medical services provided to pilgrims during this period.

### Data collection

The audit tool used in this study was adapted from an Australian audit tool designed by the Victorian State Government Department of Human Services [3]. Data were collected on standardized checklists on various infection control service items during surprise visits to the medical, pediatric, surgical, and critical care units, and the kitchens of the audited hospitals. Where satisfaction of an item was not possible by observation, a response obtained by staff questioning was accepted. The audit members comprised six infectious diseases consultants divided into three teams. Each team was assigned to review two different hospitals every year for four consecutive years.

Eleven areas of infection control service were identified for the audit, namely: hand washing, environmental cleaning, waste disposal, handling of clean linen, handling of soiled linen, standard and transmission-based precautions, single use policy, urinary catheter care, sterile wound dressing, food hygiene, and pests and animal control in clinical areas. The details of the items audited under each of these eleven areas are shown in an additional file [See Additional File 1]. The hospitals were expected to follow the guideline for isolation precautions in hospitals recommended by the Centers for Disease Control and Prevention (CDC) in 1996 [4]. Any negative or unsatisfactory finding was given a score of zero; any positive or satisfactory finding was given a score of one; any partially met finding was given half a mark. When an item was not applicable to the hospital, it was marked as ''non-applicable'' (NA). Non-applicable items were not included in the final numeric score. If an item audited in different units in the same hospital received different scores, the lowest score was taken as the final score of that item. The total maximum score for handwashing was 21 marks, for environmental cleaning and sanitation, 19 marks, for waste disposal, 20 marks, for handling and storage of clean linen, 5 marks, for handling and storage of soiled linen, 10 marks, for standard and transmission-based precautions (contact, droplet, and airborne precautions), 32 marks, for single use policy, 3 marks, for urinary catheter drainage, 5 marks, for sterile wound dressing, 11 marks, for food hygiene, 13 marks, and for vector control in clinical areas, 6 marks. The percentage score of any area was calculated as the total marks obtained for the different items audited in the area (the numerator), divided by the total marks of the audited items (the denominator), and multiplied by a hundred.

In addition to the aforementioned areas, the audit included collecting information about the presence of an infection control committee in the hospital, whether the committee met regularly, whether the meetings were appropriately minuted, the number of infection control team staff, the presence of educational activities on infection control directed to health care workers, and the presence of surveillance data.

### Feedback to the audited hospitals

The result of the audit for each hospital was confidentially sent to it within four weeks after the end of Hajj. Hospitals were expected to utilize the results of these audits to improve their infection control services.

## Results

**Figure 1:**
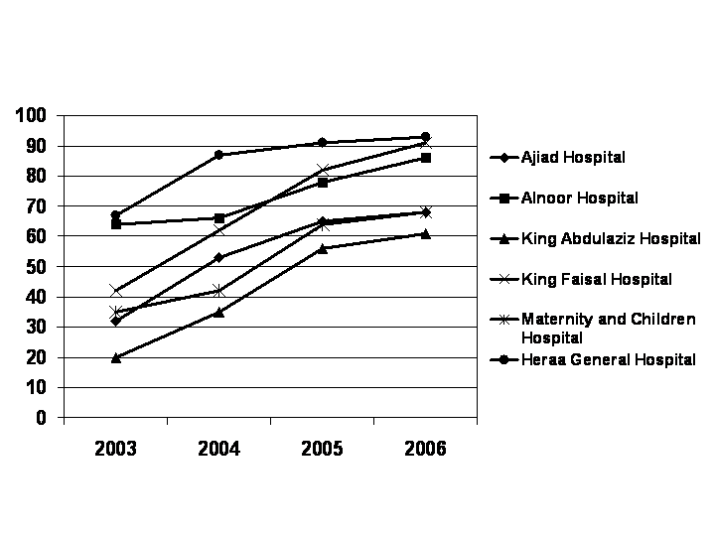
Trends of the annual total percent scores on eleven audited infection control areas in six community hospitals in Makkah, Saudi Arabia for four consecutive years (2003–2006).

On average, 12–16 hours were required by any of the three teams to complete the audit of each hospital. Tables 1,2,3,4,5,6 summarize the results of the audits for the six hospitals for four consecutive years. Figure 1 depicts the trends in the annual total percent scores on the eleven audited infection control items for the six hospitals. HGH had the highest score in the four audits with further improvement observed every year. The infection control unit in HGH was chaired by an active and well qualified microbiologist who was able to utilize and take advantage of the results of the audits to further improve the infection control service. Further, the hospital administration was extremely supportive to the infection control unit and the infection control committee. Any recommendations to improve the infection control practice were given the highest priority by the hospital administration. KFH also had remarkable improvement of its audit score with approximately 20% increment in the total average score every year in the first three years. The main reason for the improvement observed in KFH was also the remarkable administrative commitment and dedication to resolve the deficiencies reported in the audits and to follow the audit's recommendations. The other four hospitals also showed steady, albeit less remarkable, improvements in all aspects of the audit.

**Table 1:** Results of the infection control audits for Ajiad Hospital for four consecutive years

Year	2003	2004	2005	2006
Total number of beds	140	140	140	140
Number of critical care beds	18	18	18	18
Infection control committee in place	No	No	Yes	Yes
Infection control committee constituted of representative members	NA	NA	Yes	Yes
Regular infection control committee meetings	NA	NA	Yes	Yes
Adequate infection control committee minutes	NA	NA	Yes	Yes
Infection control unit	Yes	Yes	Yes	Yes
Number of infection control team staff	2	4	5	5
Nurses	1	2	2	2
Environmental inspectors	1	1	1	1
Doctors	0	1	1	1
Microbiologists	0	0	1	1
Educational activities	No	No	Yes	Yes
Surveillance system	No	No	Yes	Yes
Area audited	Percent scores			
Handwashing	16	61	59	77
Environmental cleaning	50	56	62	58
Waste disposal	0	50	82	82
Handling of clean linen	0	60	60	70
Handling of soiled linen	0	50	70	60
Standard and transmission based precautions	29	36	50	56
Single use policy	33	33	67	50
Urinary catheter care	40	40	60	80
Sterile wound dressing	64	64	73	69
Food hygiene	54	69	69	83
Pests and animal control in clinical areas	67	67	67	67
Average total percent score	32	53	65	68

NA: not applicable.

**Table 2:** Results of the infection control audits for Alnoor Specialist Hospital for four consecutive years

Year	2003	2004	2005	2006
Total number of beds	650	557	557	557
Number of critical care beds	43	43	43	43
Infection control committee in place	Yes	Yes	Yes	Yes
Infection control committee constituted of representative members	Yes	Yes	Yes	Yes
Regular infection control committee meetings	Yes	Yes	Yes	Yes
Adequate infection control committee minutes	Yes	Yes	Yes	Yes
Infection control unit	Yes	Yes	Yes	Yes
Number of infection control team staff	4	4	7	7
Nurses	3	3	2	2
Environmental inspectors	0	0	3	3
Doctors	0	0	1	1
Microbiologists	1	1	1	1
Educational activities	No	No	Yes	Yes
Surveillance system	No	No	Yes	Yes
Area audited	Percent scores			
Handwashing	61	61	79	91
Environmental cleaning	78	78	78	90
Waste disposal	62	73	86	95
Handling of clean linen	60	60	60	90
Handling of soiled linen	50	50	60	78
Standard and transmission based precautions	45	48	77	83
Single use policy	33	33	100	83
Urinary catheter care	80	80	80	80
Sterile wound dressing	64	73	82	92
Food hygiene	67	67	75	73
Pests and animal control in clinical areas	100	100	83	92
Average total percent score	64	66	78	86

**Table 3:** Results of the infection control audits for King Abdulaziz Hospital for four consecutive years

Year	2003	2004	2005	2006
Total number of beds	272	272	272	272
Number of critical care beds	30	30	30	30
Infection control committee in place	No	No	No	Yes
Infection control committee constituted of representative members	NA	NA	NA	Yes
Regular infection control committee meetings	NA	NA	NA	Yes
Adequate infection control committee minutes	NA	NA	NA	Yes
Infection control unit	No	No	Yes	Yes
Number of infection control team staff	1	1	4	6
Nurses	1	1	2	2
Environmental inspectors	0	0	1	2
Doctors	0	0	1	2
Microbiologists	0	0	0	0
Educational activities	No	No	Yes	Yes
Surveillance system	No	No	Yes	Yes
Area audited	Percent scores			
Handwashing	10	32	57	57
Environmental cleaning	16	22	54	58
Waste disposal	5	18	50	50
Handling of clean linen	0	0	40	40
Handling of soiled linen	0	40	50	44
Standard and transmission based precautions	29	29	59	72
Single use policy	33	33	67	67
Urinary catheter care	60	80	60	67
Sterile wound dressing	0	45	80	70
Food hygiene	54	69	69	62
Pests and animal control in clinical areas	17	17	33	83
Average total percent score	20	35	56	61

NA: not applicable.

**Table 4:** Results of the infection control audits for King Faisal Hospital for four consecutive years

Year	2003	2004	2005	2006
Total number of beds	207	207	207	207
Number of critical care beds	22	22	22	22
Infection control committee in place	Yes	Yes	Yes	Yes
Infection control committee constituted of representative members	Yes	Yes	Yes	Yes
Regular infection control committee meetings	Yes	Yes	Yes	Yes
Adequate infection control committee minutes	Yes	Yes	Yes	Yes
Infection control unit	Yes	Yes	Yes	Yes
Number of infection control team staff	2	2	3	3
Nurses	2	2	2	2
Environmental inspectors	0	0	0	0
Doctors	0	0	1	1
Microbiologists	0	0	0	0
Educational activities	No	No	Yes	Yes
Surveillance system	No	Yes	Yes	Yes
Area audited	Percent scores			
Handwashing	30	57	76	95
Environmental cleaning	69	77	82	93
Waste disposal	36	50	91	96
Handling of clean linen	40	60	80	100
Handling of soiled linen	30	60	70	89
Standard and transmission based precautions	29	57	88	78
Single use policy	33	33	67	83
Urinary catheter care	80	80	80	100
Sterile wound dressing	44	67	78	100
Food hygiene	8	62	85	100
Pests and animal control in clinical areas	67	83	100	67
Average total percent score	42	62	82	91

**Table 5:** Results of the infection control audits for the Maternity and Children Hospital for four consecutive years

Year	2003	2004	2005	2006
Total number of beds	250	250	250	250
Number of critical care beds	24	24	24	24
Infection control committee in place	Yes	Yes	Yes	Yes
Infection control committee constituted of representative members	Yes	Yes	Yes	Yes
Regular infection control committee meetings	No	No	Yes	Yes
Adequate infection control committee minutes	No	No	Yes	Yes
Infection control unit	No	No	Yes	Yes
Number of infection control team staff	2	2	4	4
Nurses	2	2	2	2
Environmental inspectors	0	0	0	0
Doctors	0	0	2	2
Microbiologists	0	0	0	0
Educational activities	No	No	Yes	Yes
Surveillance system	No	No	Yes	Yes
Area audited	Percent scores			
Handwashing	48	65	78	68
Environmental cleaning	33	33	62	58
Waste disposal	27	41	59	61
Handling of clean linen	20	20	40	60
Handling of soiled linen	0	10	33	57
Standard and transmission based precautions	29	32	76	89
Single use policy	33	33	67	75
Urinary catheter care	60	80	100	60
Sterile wound dressing	55	55	91	64
Food hygiene	46	62	69	75
Pests and animal control in clinical areas	33	33	33	83
Average total percent score	35	42	64	68

**Table 6:** Results of the infection control audits for Heraa General Hospital for four consecutive years

Year	2003	2004	2005	2006
Total number of beds	261	261	261	261
Number of critical care beds	21	21	21	21
Infection control committee in place	Yes	Yes	Yes	Yes
Infection control committee constituted of representative members	Yes	Yes	Yes	Yes
Regular infection control committee meetings	Yes	Yes	Yes	Yes
Adequate infection control committee minutes	Yes	Yes	Yes	Yes
Infection control unit	Yes	Yes	Yes	Yes
Number of infection control team staff	4	4	4	4
Nurses	2	2	2	2
Environmental inspectors	1	1	1	1
Doctors	0	0	0	0
Microbiologists	1	1	1	1
Educational activities	Yes	Yes	Yes	Yes
Surveillance system	No	Yes	Yes	Yes
Area audited	Percent scores			
Handwashing	52	83	83	93
Environmental cleaning	94	94	83	84
Waste disposal	60	91	96	98
Handling of clean linen	75	75	100	100
Handling of soiled linen	90	90	90	90
Standard and transmission based precautions	42	86	91	89
Single use policy	33	67	100	100
Urinary catheter care	80	100	100	100
Sterile wound dressing	82	91	100	100
Food hygiene	50	83	92	96
Pests and animal control in clinical areas	83	100	67	75
Average total percent score	67	87	91	93

Handwashing scored low in all hospitals in the first audit but it markedly improved in the subsequent audits mainly due to the use of waterless alcohol handrub as an alternative to handwashing with water and soap. Another important infection control deficiency observed in most of the hospitals was the limited understanding and implementation of standard and transmission-based precautions. All hospitals were following old isolation guidelines when the first audit was conducted in year 2003. The new isolation guidelines recommended by the CDC were implemented in the subsequent years but the improvement was somewhat slow as the process of educating and training HCWs on these new concepts of isolation was rather long.

The rate of improvement of the audit score in the first three years was somewhat faster than that for the fourth year (Figure 1). The initial fast improvement was mainly attributed to resolving infection control deficiencies that required no extra-resources. The slower improvement noticed subsequently was attributable to infetion control items that required extra-resources to be resolved or improved as it took hospitals one to three years to get such extra-resources secured.

## Discussion

Clinical audit is a quality improvement process that seeks to improve patient care and outcomes through systematic review of care against explicit criteria and the implementation of change [5]. The so called, audit cycle, comprises five basic stages: choosing a topic, specifying appropriate practice standards, testing actual practice against these standards (data collection), correcting practice where it falls short, and finally, re-auditing to confirm that standards are met [6,7]. Attainment of standards may only be achieved after several rounds of the audit cycle [7].

Hospital infection control is a good subject for audit as it affects patient care, quality of life and clinical outcomes [7]. Additionally, evidence-based standards of practice have been developed [6-10]. It is now accepted that audit is a key function for infection control teams [7,11,12]. Audit programs should include audits of infection control policies in wards and departments, microbiological safety and cleanliness audits of the hospital environment, and audits of standard healthcare equipment [7].

The most effective strategies to prevent health care associated infections include audit of the incidence of infection, feedback of these infection rates to clinical staff, continuous infection control education programs, one infection control nurse for every 250 beds, and infection control audit for evaluating clinical practice [13]. The results of the current study confirmed the enormous positive impact of audits on infection control service and practice. All six hospitals had tangible improvements of all aspects of infection control. The improvement was most pronounced in hospitals that obtained the lowest scores in the first audit. Early feedback of the results of the audits to the concerned hospitals was essential for the hospitals to resolve the weaknesses and maintain the strengths. The availability of qualified and well trained personnel and support of the infection control services and committees by the hospital administration were the main driving forces for proper utilization of the audits' results that lead to noticeable improvement in infection control services.

Many deficiencies observed in the first audit were subsequently resolved. AGH and KAH had no infection control committees and KAH and MCH had no infection control units in the first year of the audit. Subsequently, appropriate infection control committees and units were established in these hospitals. Notably, all hospitals except HGH had no infection control educational activities when audited first. Subsequently, such activities were initiated. At the outset, all hospitals had no proper surveillance system for health care associated infections. This defect was also resolved in the subsequent years. HGH had four infection control staff (one staff per 65 beds) throughout the study period. The other five hospitals that had shortage of infection control staff (nurses, environmental inspectors, doctors, and/or microbiologists) managed to recruit more staff over the study period. AGH increased the number of infection control staff from two to five staff (one staff per 28 beds), NSH, from four to seven staff (one staff per 80 beds), KAH, from none to six staff (one staff per 45 beds), KFH, from two to three staff (one staff per 69 beds), and the MCH, from two to four staff (one staff per 65 beds). The number of infection control nurses per beds was 1/70 for AGH, 1/186 for NSH, 1/136 for KAH, 1/103 for KFH, and 1/130 each for MCH and HGH. These ratios were better than the recommended ratios for effective infection control programs [14-16].

Handwashing scored low in all hospitals in the first audit but it markedly improved in the subsequent audits mainly due to the use of waterless alcohol handrub as an alternative to handwashing with water and soap. Other observational studies indicate that, eventhough handwashing is known to be the single most important means of preventing the spread of micro-organisms in the healthcare setting, adherence of health care workers to handwashing practice is low with mean baseline rates of 5%–81%, and an overall average of 40% [17]. A recent study from Saudi Arabia showed that the overall frequency of handwashing after patient contact among health care workers in medical and surgical wards in a tertiary care center in Riyadh was only 23.7% [18]. Reported risk factors for poor adherence to recommended hand hygiene practices include handwashing agents causing irritation and dryness, sinks that are inadequate in number or inconveniently located, and lack of soap and paper towels [17,19]. Easy access to hand hygiene supplies, whether sink, soap, medicated detergent, or alcohol-based hand-rub solution, is essential for optimal adherence to hand hygiene recommendations. In this study, improvement of hand hygiene score in the audited hospitals was mainly due to the use of alcohol handrub as an alternative to hand washing with water and soap. Providing easy access to hand hygiene materials is achievable in the majority of health-care facilities [19]. In contrast to sinks used for handwashing or antiseptic handwash, dispensers for alcohol-based hand rubs do not require plumbing and can be made available adjacent to each patient's bed and at many other locations in patient care areas. Further, using alcohol-based hand rubs may be a better option than conventional handwashing with plain or antiseptic soap and water as they require less time, act faster, and irritate hands less often [20-24]. Additionally, their use was shown to lead to a sustained improvement in adherence to hand hygiene and decreased infection rates and to be cost effective [25]. The deficiencies observed in the other items of infection control (environmental cleaning, waste disposal, handling of clean linen, handling of soiled linen, standard and transmission based precautions, single use policy, urinary catheter care, sterile wound dressing, food hygiene, and pests and animal control in clinical areas) were likewise resolved or improved over the study period in the audited hospitals.

Previous studies evaluating the effects of audit and feedback on professional practice and health care outcomes showed variable results (25–26). Cochrane reviews of these studies conclude that audit and feedback yield a small to modest improvement in the practice of health care professionals, regardless of whether they are used alone or in concert with other forms of intervention (25–26). The relative effectiveness of audit and feedback is likely to be greater when baseline adherence to recommended practice is low and when feedback is delivered more intensively (25). These reviews were hampered by the fact that many published studies are too small, not rigorously designed and lack detailed descriptions of interventions (6). Our study demonstrated a substantial positive impact of audit and feedback on infection control practice. There were, however, some limitations of this study. The eleven areas audited in this study were not all-inclusive. Several other areas were not included in the audits such as infection control in the hemodialysis units, central sterile supply departments, operating, delivery, and emergency rooms, laboratories, pharmacies, and outpatient departments. Further, the impact of the improvement of infection control practice on the rate of health-care associated infections in the audited hospitals was not assessed in this study. It is conceivable, however, that such improvement in infection control practice would have had a significant positive impact on the rate of health-care associated infections as the audits included items that are considered to be evidence-based standards of practice in infection control to prevent health-care associated infections [6-10].

In conclusion, regular hospital infection control audits lead to significant improvement of infection control practice and hence improvement of patient safety. Infection control should be a top priority in hospitals. There is a need to build a rigorous infection control audit into hospitals' ongoing monitoring and reporting to the MOH and to provide these hospitals with feed back on such audits to continuously strengthen the safety standards for patients, visitors, and employees.

## Competing interests

The author(s) declare that they have no competing interests.

## Authors' contributions

TAM designed the study and the audit tool, participated in auditing the hospitals, analyzed the data, and wrote the manuscript. AMB, MAH, TAA, AOT, and AHI participated in designing the audit tool and auditing the hospitals. All authors read and approved the final manuscript.

## Pre-publication history

The pre-publication history for this paper can be accessed here:



## Supplementary Material

Additional File 1 Infection control items audited. The table shows the details of the infection control items audited Click here for file
