# Supplementary material for: Steady improvement of infection control services in six community hospitals in Makkah following annual audits during Hajj for four consecutive years
Source: BMC Infect Dis. 2006 Aug 25;6:135. doi: 10.1186/1471-2334-6-135 (PMC1590039; doi:10.1186/1471-2334-6-135)
Supplement: Additional File 1 — Infection control items audited. The table shows the details of the infection control items audited [file 1471-2334-6-135-S1.doc]

| **Audited items** |
| --- |
| Handwashing |
| The presence of adequate number of handwashing facilities for staff |
| The presence of soap on basins for staff use |
| Hands were washed under warm, running water |
| The use of a plain soap for handwashing |
| Washing of all areas of both hands using friction |
| Jewellery other than wedding ring was not worn by staff |
| Area under wedding ring was washed, |
| Handwashing time was at least 15 seconds |
| Hands were rinsed free of soap |
| Hands were dried using paper towel, including the area under the ring |
| Alcohol for waterless handrub was used between patient contacts as an alternative  when handwashing facilities were not adequate |
| Presence of a poster depicting good handwashing technique at hand basins |
| Handwashing technique was reinforced at the staff induction programs |
| The need to wash hands after glove removal was recognized by staff |
| Reusable towels were not in use and paper towels were available for drying the hands  after washing them with soap and water |
| Used paper towels were disposed of appropriately |
| The availability of a good quality, clearly labeled moisturizer at staff handwashing  facilities |
| Hand moisturizers were used routinely, as indicated |
| Staff fingernails were clean and short |
| The presence of a procedure for staff to notify the infection control department  regarding personal hand problems such as break-down, rashes, and paronychia |
| Disposable gloves were worn for activities in which hand contamination was possible |
| **Environmental cleaning and sanitation** |
| The availability of a documented procedure for all cleaning activities within clinical  areas (including baths after patients’ use, blood pressure cuffs, sponge bowls,  commode chairs, lifting slings, and cleaning equipment), |
| Surfaces and equipment were free from dust, spills, and grime (including hard and  soft floors, patients’ over-bed tables, bed-side lockers, medical equipment,  bathrooms, and ward kitchens), |
| Cleaning equipment was color-coded to identify appropriate area of use, |
| Equipment for sanitation and decontamination was functioning effectively during the  preceding four weeks and quickly repaired if malfunctioning (including pan/urinal  sinister, utensil washers, sluices, staff hand basins, and toilets). |
| **Waste disposal** |
| The availability of a documented procedure and flowchart to guide staff in waste  disposal including infectious and ordinary wastes |
| Waste flowchart was posted in utility rooms and waste storage rooms |
| Details of waste disposal flows were included in the staff induction programs |
| Foot-operated basins were available for infectious waste |
| Waste bags were securely sealed when filled |
| Full waste bags were only kept in designated areas |
| Infectious waste and other scheduled waste were kept under secured conditions |
| Rooms where infectious waste kept were signposted with warning symbols |
| Sealed infectious waste bags were traceable to the clinical area of origin |
| Sharp boxes used for the reception of all sharps were available in all areas where  sharps were generated |
| Sharp boxes were securely sealed when two-thirds full |
| Sharp boxes contained no protruding sharps |
| Sharp boxes were secure from public interference |
| Sharp boxes were traceable to the clinical area of origin |
| Information regarding sharps disposal was provided within the staff induction  programs |
| An accessible procedure for containment and decontamination of biological spills  (e.g.: blood) was available |
| The procedure for dealing with biological spills was detailed in the staff induction  programs |
| A biological spill kit was available in clinical areas |
| The spill kit was complete and functional |
| The staff were aware of the location of the spill kit and its mode of use |
| **Handling and storage of clean linen** |
| Clean linen were transported to and stored in the clinical area in such a manner that  exposure to dust and moisture was prevented |
| Clean linen were physically separated from soiled line |
| Unnecessary supplies of clean linen were not taken to patients’ rooms or placed on  patients’ furnishings |
| The presence of a mechanism for staff to report if clean linen were not of adequate  standard |
| Clean linen were not kept in an area housing cleaning equipment |
| **Handling and storage of soiled linen** |
| Soiled linen skips were available in adequate number where bed stripping activities  occurred |
| Soiled linen were not carried by hand more than a few meters |
| Soiled linen skips had a lid and were foot operated |
| Soiled linen bags were closed off when two-thirds full |
| Gloves were worn when handling heavily soiled linen |
| A mechanism for segregating and color coding foul, ordinary, and infectious linen  was present |
| The mechanism for segregating different soiled linen was reinforced in the staff  induction programs |
| Soiled linen capable of leakage were placed in bags with impervious liners |
| Soiled linen bags in designated areas were secure from public access |
| Staff washed hands following soiled linen handling |
| **Standard and transmission-based precautions** |
| Standard precautions |
| A procedure for standard precautions was present in clinical areas |
| Standard precautions were reinforced in the staff induction programs |
| Disposable gloves, aprons, and eye shields were available in clinical areas |
| Containerized pathology specimens were placed in leak proof transit bags for  transport |
| Appropriate protective clothing was worn when contact with body fluids was  expected |
| Suitable disposable gloves were available for staff suffering from latex allergy |
| Hands were washed after glove removal |
| Contact precautions |
| The use of single room or cohorting, |
| Gloves and gowns were available by the door outside the room, gloves were worn by  staff when entering the room, |
| Gowns were worn by staff when entering the room, |
| Masks were not used unless the organism of concern was present in the sputum, |
| Gloves and gowns were removed and discarded in a yellow bag inside the room  before exiting, |
| Hands were washed after removal of the gowns and gloves, |
| A contact precautions sign was posted on the wall by the door (the door could be  kept open) |
| Droplet precautions |
| The use of single room or cohorting, |
| Surgical masks were available by the door outside the room, |
| Masks were worn by staff when entering the room, |
| Gowns and gloves were not used unless the organism was also transmitted by  contact, |
| Mask was removed and discarded in a yellow bag inside the room before exiting |
| Hands were washed after removing the mask, |
| A droplet precautions sign was posted on the wall by the door (the door could be kept  open). |
| Airborne precautions |
| The use of single negative pressure room for isolation, |
| The pressure inside the room was confirmed to be negative (tested with a tissue) |
| A monitoring device for air exchanges (6-12 times per hour) was present |
| High filtration masks were available by the door outside the room |
| High filtration masks were used by staff when entering the room |
| Gowns and gloves were not used unless the organism was also transmitted by contact |
| Mask was removed and discarded in a yellow bag inside the room before exiting |
| Hands were washed after removing the mask |
| An airborne precautions sign was posted on the door (not on the wall by the door) |
| The door of the isolation room was kept closed |
| **Single use policy** |
| The presence of a single use policy |
| Multi-dose vials in use in clinical areas were labeled with the hour and date opened |
| Disposable items were not reprocessed or reused |
| **Urinary catheter drainage** |
| Disposable gloves were worn for emptying drainage bag |
| Drainage bags were above the floor level |
| A disinfected jug or disposable container was used for emptying the urinary bags |
| Catheter urine specimens were obtained by the aseptic swab/puncture/syringe method |
| The valve in the drainage bag was actually preventing leakage |
| **Sterile wound dressing** |
| Sterile supplies were assembled totally prior to commencing aseptic techniques |
| Hands were washed prior to opening sterile supplies |
| Sterile supplies were opened aseptically |
| Procedural hand washing was adequate |
| Sterile gloves were donned aseptically |
| A soiled dressing was removed and discarded appropriately with removal forceps |
| Wounds were swabbed using aseptic techniques |
| Sterile dressing was applied aseptically |
| Used-items were disposed of appropriately |
| Gloves were removed in a manner that did not result in hand contamination |
| Staff washed hands after removing the gloves |
| **Food hygiene** |
| Hand washing basins were available in the kitchen |
| Disposable gloves were available in the kitchen |
| The kitchen dishwasher functioned effectively over the preceding four weeks |
| The kitchen staff completed a food-handlers educational course |
| The kitchen cleaning equipment was color-coded |
| The correct color-coded equipment was actually used |
| The cold food items were kept under a temp not greater than 5^O^C |
| The hot food items were kept under a temp not less than 65^O^C |
| All plated food placed in the refrigerator were covered with transparent wrap |
| Hand washing occurred prior to food preparation |
| Disposable or single use toweling was used for cleaning |
| The kitchen had a cleaning schedule |
| There was evidence that the kitchen cleaning schedule was followed |
| **Vector control in clinical areas** |
| Windows had intact fixed screens |
| Mosquitoes were not seen |
| Flies were not seen |
| Cockroach were not seen |
| Rats were not seen |
| Cats were not seen |
